# Supplementary material for: COZOID: contact zone identifier for visual analysis of protein-protein interactions
Source: BMC Bioinformatics. 2018 Apr 6;19:125. doi: 10.1186/s12859-018-2113-6 (PMC5889581; doi:10.1186/s12859-018-2113-6)
Supplement: Supplementary file 2 — Software build. Executable binary file of the software tool. (ZIP 118,784 kb) [file 12859_2018_2113_MOESM2_ESM.zip › cozoid/java/docs/beansbinding-1.2.1-doc.zip!/org/jdesktop/observablecollections/ObservableCollections.ObservableListHelper.html]

ObservableCollections.ObservableListHelper (Beans Binding)


---


|  |  |  |
| --- | --- | --- |
| **Overview** \| **Package** \| **Class** \| **Use** \| **Tree** \| **Deprecated** \| **Index** \| **Help** | |  |
| **PREV CLASS**   **NEXT CLASS** | **FRAMES**    **NO FRAMES**     **All Classes** |  |
| SUMMARY: NESTED \| FIELD \| CONSTR \| METHOD | DETAIL: FIELD \| CONSTR \| METHOD |  |


---


## org.jdesktop.observablecollections Class ObservableCollections.ObservableListHelper<E>

```
java.lang.Object
  org.jdesktop.observablecollections.ObservableCollections.ObservableListHelper<E>
```

**Enclosing class:**: ObservableCollections

---

``` public static final class ObservableCollections.ObservableListHelper<E> extends java.lang.Object ```

`ObservableListHelper` is created by `observableListHelper`,
and useful when changes to individual elements of the list can be
tracked.

**See Also:**: `ObservableCollections.observableListHelper(java.util.List)`

---

| **Method Summary** | |
| --- | --- |
| `void` | `fireElementChanged(int index)`             Sends notification that the element at the specified index has changed. |
| `ObservableList<E>` | `getObservableList()`             Returns the `ObservableList`. |

**Methods inherited from class java.lang.Object**

`clone, equals, finalize, getClass, hashCode, notify, notifyAll, toString, wait, wait, wait`

**Method Detail**

### getObservableList

```
public ObservableList<E> getObservableList()
```

:   Returns the `ObservableList`.

    :   **Returns:**: the observable list

---


### fireElementChanged

```
public void fireElementChanged(int index)
```

:   Sends notification that the element at the specified index
    has changed.

    :   **Parameters:**: `index` - the index of the element that has changed **Throws:**: `java.lang.ArrayIndexOutOfBoundsException` - if index is outside the range of the `List` (`< 0 || >= size`)


---


|  |  |  |
| --- | --- | --- |
| **Overview** \| **Package** \| **Class** \| **Use** \| **Tree** \| **Deprecated** \| **Index** \| **Help** | |  |
| **PREV CLASS**   **NEXT CLASS** | **FRAMES**    **NO FRAMES**     **All Classes** |  |
| SUMMARY: NESTED \| FIELD \| CONSTR \| METHOD | DETAIL: FIELD \| CONSTR \| METHOD |  |


---
